# Analysis of multi-lineage gene expression dynamics during primordial germ cell induction from human induced pluripotent stem cells

**DOI:** 10.1186/s13287-020-01620-y

**Published:** 2020-03-04

**Authors:** Fang Fang, Zili Li, Qian Zhao, Chengliang Xiong, Ke Ni

**Affiliations:** 1 Department of Obstetrics and Gynecology, Union Hospital, Tongji Medical College, Huazhong University of Science and Technology, 1277 Jiefang Avenue, Wuhan, 430022 China; 2 Institute of Reproductive Health, Tongji Medical College, Huazhong University of Science and Technology, 13 Hangkong Road, Wuhan, 430030 China; 3 Wuhan Tongji Reproductive Medicine Hospital, 128 Sanyang Road, Wuhan, 430013 China; 4 School of Basic Medical Sciences, Wuhan University, 115 Donghu Road, Wuhan, 430071 China

**Keywords:** Primordial germ cells, Induced pluripotent stem cells, BMP, EpCAM, INTEGRINα6, Lineage genes

## Abstract

**Background:**

In mammals, specification of primordial germ cells (PGCs) is established in the early post­implantation embryo. The bone morphogenetic protein (BMP)-SMAD and WNT3-β-catenin signaling initiate the gene regulatory network for PGC specification. The activation of SOX17-BLIMP1 axis is critical for human PGC program. Moreover, EpCAM and INTEGRINα6 were identified as surface markers of human PGC-like cells (PGCLCs) recently. However, the signaling mechanism for PGC specification in non­rodent mammals remains to be clarified.

**Methods:**

We differentiated human induced pluripotent stem cells (hiPSCs) into PGCLCs in vitro in response to Activin A and BMP4. The percentage of EpCAM/INTEGRINα6 double-positive cells (PGCLCs) was analyzed by flow cytometry. The expression of PGC genes was evaluated by qRT-PCR and immunofluorescence. The expression dynamic of multi-lineage genes during the differentiation process was evaluated by qRT-PCR.

**Results:**

Under the stimulation for PGCLC induction, the embryoids derived from hiPSCs initiated significant upregulation of the early PGC genes (BLIMP1, TFAP2C, and NANOS3), but maintained low or no levels of DPPA3 and late PGC genes (DAZL and DDX4). The percentage of EpCAM/INTEGRINα6 double-positive PGCLCs reached the highest at day 6 of induction. After pre-induction, the incipient mesoderm-like cells (iMeLCs) upregulated most of the mesoderm genes (EOMES, T, MSXI, RUNX2, and MIXL1). The differentiating embryoids showed high levels of key pluripotency genes, OCT4 and NANOG, but became negative for SOX2. In contrast to iMeLCs, the differentiating embryoids downregulated mesoderm genes RUNX2 and EOMES, and ectoderm gene PAX6, but increased the expression of endoderm gene SOX17.

**Conclusions:**

During PGCLC induction process in vitro, the differentiating embryoids not only activated the PGC-related genes, but also displayed complex regulation of pluripotency genes and multi-lineage genes. These results would be meaningful for future research investigating the regulation of human early germ line development.

## Introduction

The germ cell lineage is the source of totipotency and transmits genetic and epigenetic information across generations. In mammals, primordial germ cells (PGCs) are the founder cells of the germ cell lineage and emerge at early stage of post-implantation development. The PGCs undergo a number of complex developmental events, including repression of somatic programs, (re)acquisition of potential pluripotency, and epigenetic reprogramming, and finally initiate oogenesis or spermatogenesis to form gametes [1]. Currently, the mechanism of PGC specification has been extensively investigated in mice, which provides insight into mammalian development. In mice, the bone morphogenetic protein (BMP) and WNT signals from extra-embryonic tissues induce the expression of several PGC fate regulators in a few germ line competent cells [2–4]. Particularly, a tripartite transcription factor network of PR domain zinc finger protein 1 (PRDM1, also known as BLIMP1), PRDM14, and transcription factor AP-2 gamma (TFAP2C) represses the somatic fate and promotes the mouse PGC specification [5].

By contrast, the knowledge about human PGC specification is relatively limited because of the technical and ethical obstacles to obtain germ line cells from early human embryos. Notably, in the last decade, generation of germ cells from pluripotent stem cells (embryonic stem cells, ESCs, and induced pluripotent stem cells, iPSCs) has provided a surrogate model for germ line development in vitro*.* In mice, ESCs and iPSCs with naive pluripotency could be induced into primordial germ cell-like cells (PGCLCs) through epiblast-like cells (EpiLCs) [6]. However, human ESCs (hESCs) and iPSCs (hiPSCs) are considered to show a primed pluripotency with limited potential for germ cell fate and respond poorly to the method used for mouse PGCLC induction [7–9]. Accordingly, Gafni et al. established defined conditions to generate human naive pluripotent stem cells from primed hESCs and hiPSCs [10]. Moreover, Irie et al. developed an approach for human PGCLC (hPGCLCs) specification from the ground state hESCs and hiPSCs [7]. Strikingly, hPGCLCs are robustly induced in vitro from hiPSCs in a primed pluripotent state through incipient mesoderm-like cells (iMeLCs) [11]. Based on these differentiation models, several key regulators of human PGC (hPGC) fate as well as the regulation network they formed were clarified, including EOMES and SOX17 [7, 12].

The mammalian germline is set aside from somatic lineages in early post-implantation embryos [1]. During the in vitro hPGCLC specification process, only some of the pluripotent stem cells respond to the induction signals, and the other cells still spontaneously differentiated into somatic lineages. Here, we set out to differentiate hiPSCs into hPGCLCs in vitro with the iMeLC method and determine the expression dynamics of multi-lineage genes, in order to uncover new clues for cell fate decision.

## Materials and methods

### Culture of hiPSCs

Human fibroblasts were isolated from foreskin of three volunteers at Wuhan Tongji Reproductive Medicine Hospital with written informed consent. The fibroblasts were reprogrammed with the Yamanaka KOSM (KLF4, OCT4, SOX2, and c-MYC) transcriptional factors using the lentivirus vectors. The derived hiPSCs were maintained in mTeSR1 medium (Stem Cell Technologies) on Matrigel (Corning)-coated dishes. The medium was changed every day. Cells were passaged every 3 to 5 days using Accutase (Life Technologies). For single-cell dissociation, the cells were treated with 1 to 1 mixture of TrypLE Select (Life Technologies) and 0.5 mM EDTA/PBS. Ten micrometers of a ROCK inhibitor (Y-27632, TOCRIS bioscience) was added for 24 h after passaging.

### Induction of hPGCLCs

For pre-induction, hiPSCs were dissociated with 0.5 mM EDTA/PBS, and 3× 10^5^ cells per well were plated on Matrigel-coated 12-well plates in GK15 medium (G-MEM [Thermo Fisher] supplemented with 15% KSR [Thermo Fisher], 0.1 mM NEAA [Thermo Fisher], 2 mM L-glutamine [Thermo Fisher], 1 mM sodium pyruvate [Thermo Fisher], 0.1 mM 2-mercaptoethanol [Sigma]) containing 3 μM CHIR (Selleck Chemicals), 50 ng/ml Activin A (PEPRO TECH), and 10 μM ROCK inhibitor (Y-27632, TOCRIS bioscience). After 2 days of pre-induction, the cells were dissociated with Accutase (Thermo Fisher) and plated into ultra-low cell attachment U-bottom 96-well plates (Corning) at a density of 2000–4000 cells per well to form embryoid bodies in 200 μl of GK15 medium containing 200 ng/ml BMP4 (R&D Systems), 20 ng/ml human LIF (R&D Systems), 100 ng/ml SCF (R&D Systems), 50 ng/ml EGF (R&D Systems), and 10 μM ROCK inhibitor (Y-27632, TOCRIS bioscience). H1 hESC was used as a control for PGC induction.

### Flow cytometry

The floating embryoid bodies were dissociated with 0.05%Trypsin-EDTA/PBS for 15 min at 37 °C. After washing with PBS, the cell suspension was filtered by cell strainer to remove cell clumps and then subjected to centrifugation. Then, the dissociated cells were stained with PE-conjugated anti-human EpCAM (eBioscience) and FITC-conjugated anti-human INTEGRINα6 (eBioscience). The stained cells were resuspended in PBS and analyzed with a flow cytometer (Beckman, DxFLEX).

### RNA extraction and quantitative RT-PCR

Total RNA was extracted using DirectZol RNA mini-prep (Zymo research) according to manufacturer’s instructions. Reverse transcription was performed using the RevertAid First Strand cDNA synthesis kit (Thermo Fisher Scientific). The quantitative RT-PCR was performed using SYBR Premix Ex Taq II (Takara). All gene expression analyses were performed with samples from three independent differentiation experiments. Values normalized to GAPDH are shown. Primers are listed in Table 1.

**Table 1:** Primers used in this study

Gene	Gene ID	Species	Primer sequence (5′-3′)
OCT4	5460	*Homo sapiens*	Forward: GTGTTCAGCCAAAAGACCATCT Reverse: GGCCTGCATGAGGGTTTCT
SOX2	6657	*Homo sapiens*	Forward: GCCGAGTGGAAACTTTTGTCG Reverse: GGCAGCGTGTACTTATCCTTCT
KLF4	9314	*Homo sapiens*	Forward: CGGACATCAACGACGTGAG Reverse: GACGCCTTCAGCACGAACT
NANOG	79923	*Homo sapiens*	Forward: ACAACTGGCCGAAGAATAGCA Reverse: GGTTCCCAGTCGGGTTCAC
MSX1	4487	*Homo sapiens*	Forward: TCCTCAAGCTGCCAGAAGAT Reverse: TACTGCTTCTGGCGGAACTT
RUNX2	860	*Homo sapiens*	Forward: CGGCAAAATGAGCGACGTG Reverse: CACCGAGCACAGGAAGTTG
GATA6	2627	*Homo sapiens*	Forward: CCATGACTCCAACTTCCACC Reverse: ACGGAGGACGTGACTTCGGC
BLIMP1	639	*Homo sapiens*	Forward: AAACCAAAGCATCACGTTGACA Reverse: GGATGGATGGTGAGAGAAGCAA
TFAP2C	7022	*Homo sapiens*	Forward: ATTAAGAGGATGCTGGGCTCTG Reverse: CACTGTACTGCACACTCACCTT
NANOS3	342977	*Homo sapiens*	Forward: TGGCAAGGGAAGAGCTGAAATC Reverse: TTATTGAGGGCTGACTGGATGC
PRDM14	63978	*Homo sapiens*	Forward: TATCATACTGTGCACTTGGCAGAA Reverse: AGCAACTGGGACTACAGGTTTGT
SOX17	64321	*Homo sapiens*	Forward: TTCGTGTGCAAGCCTGAGAT Reverse: TAATATACCGCGGAGCTGGC
DAZL	1618	*Homo sapiens*	Forward: TGGCCCTTCTTTCAGTGACTTC Reverse: GACCCTAGGGGGCACTAGTAA
DPPA3	359787	*Homo sapiens*	Forward: AAGCCCAAAGTCAGTGAGATGA Reverse: GCTATAGCCCAACTACCTAATGC
DDX4	54514	*Homo sapiens*	Forward: TTCTTCACAAGCTCCCAATCCA Reverse: TTCTTCTCTGCATCAAAACCACA
ZFP42	132625	*Homo sapiens*	Forward: CCAGACTGGATAACAGCAAGAGC Reverse: TGCAAATTTTTCATTCTCTAGGGC
TFCP2L1	29842	*Homo sapiens*	Forward: AGCTCAAAGTTGTCCTACTGCC Reverse: TTCTAACCCAAGCACAGATCCC
MIXL1	83881	*Homo sapiens*	Forward: TGCTTTCAAAACACTCGAGGAC Reverse: GAGTGATCGAAGTAACAGGTGC
T	6862	*Homo sapiens*	Forward: AGCCAAAGACAATCAGCAGAAA Reverse: CACAAAAGGAGGGGCTTCACTA
EOMES	8320	*Homo sapiens*	Forward: AAGGGGAGAGTTTCATCATCCC Reverse: GGCGCAAGAAGAGGATGAAATAG
PAX6	5080	*Homo sapiens*	Forward: GCCAGCAACACACCTAGTCA Reverse: TGTGAGGGCTGTGTCTGTTC
GAPDH	2597	*Homo sapiens*	Forward: TGAAGGGTGGAGCCAAAAG Reverse: AGTCTTCTGGGTGGCAGTGAT

### Immunofluorescence

Cells were fixed in 4% paraformaldehyde/PBS for 15 min at room temperature and permeabilized with 1% Triton X-100/PBS (Sigma) for 10 min. After blocking with 10% donkey serum in PBS (Jackson ImmunoResearch) for 1 h at room temperature, cells were incubated with primary antibodies overnight at 4 °C, followed by incubation with appropriate fluorescent-conjugated secondary antibodies for 1 h at room temperature the next day. Primary antibodies were as follows: OCT4 (Abcam), SOX2 (Abcam), SSEA4 (Abcam), TFAP2C (Santa Cruz), PRDM14 (Abcam), and NANOS3 (Abcam). Secondary antibodies were as follows: AlexaFluor 488 conjugated donkey anti-rabbit IgG and AlexaFluor 594 conjugated donkey anti-mouse IgG (all Life Technologies). The nuclei were counterstained with DAPI (Thermo Fisher Scientific). The cells were observed with a Zeiss inverted confocal microscope.

### Statistical analyses

All of the data were presented as mean ± standard deviation (SD). Statistical analyses were performed using the Student’s *t* test or one-way analysis of variance (ANOVA) with SPSS 17.0 software. A *p* value less than 0.05 was considered to be statistically significant.

## Results

### Differentiation of hiPSCs into PGCLCs in vitro

**Fig. 1:**
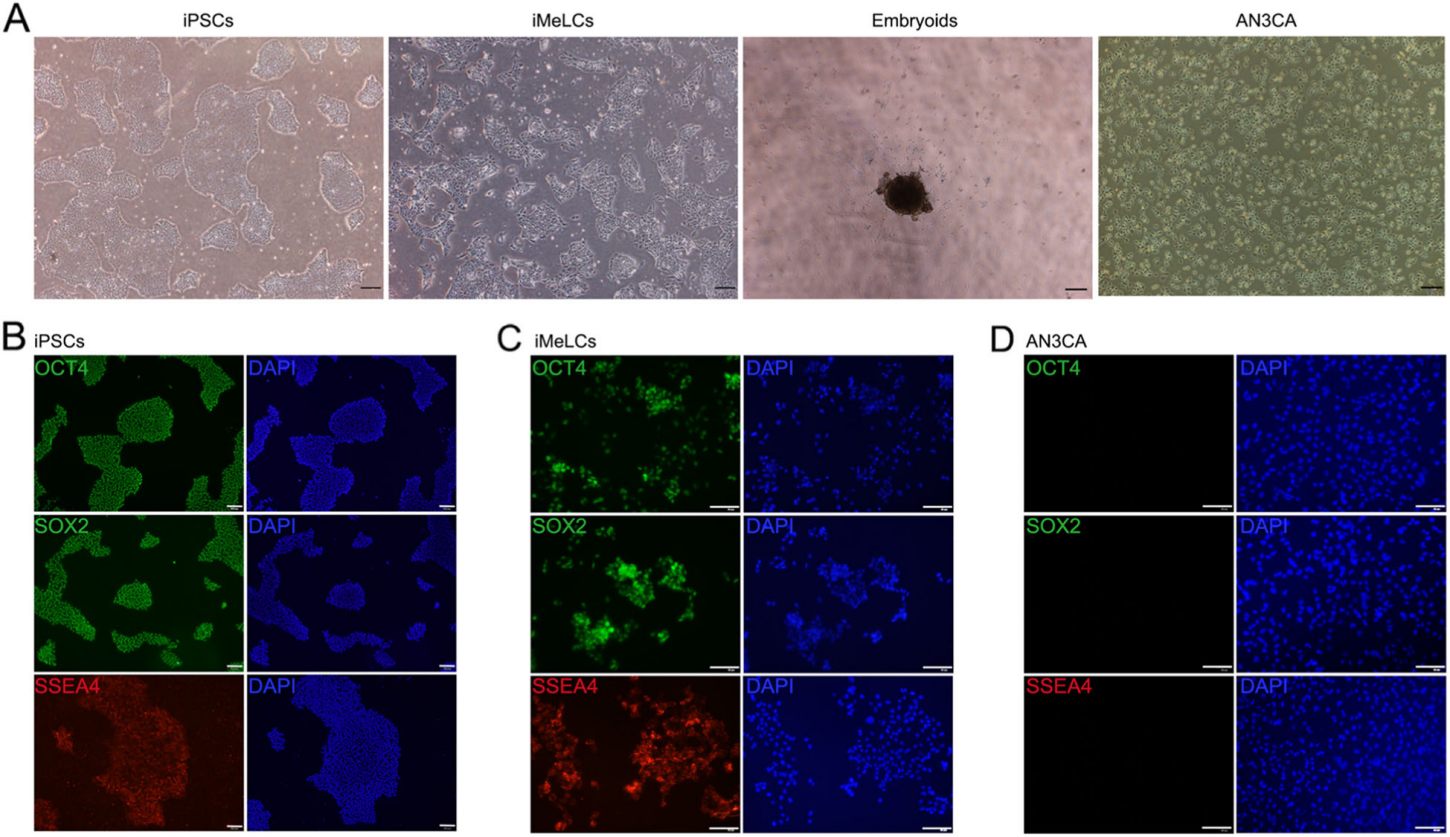
Characterization of hiPSC, iMeLCs, and embryoids. **a** Typical morphology of hiPSCs, iMeLCs, and embryoids. Scale bar, 200 μm. **b**–**d** Immunofluorescence analysis for pluripotency markers of hiPSCs (**b**) and iMeLCs (**c**). The uterine tumor cells, AN3CA, were used as negative control (**d**). Scale bar, 100 μm

**Fig. 2:**
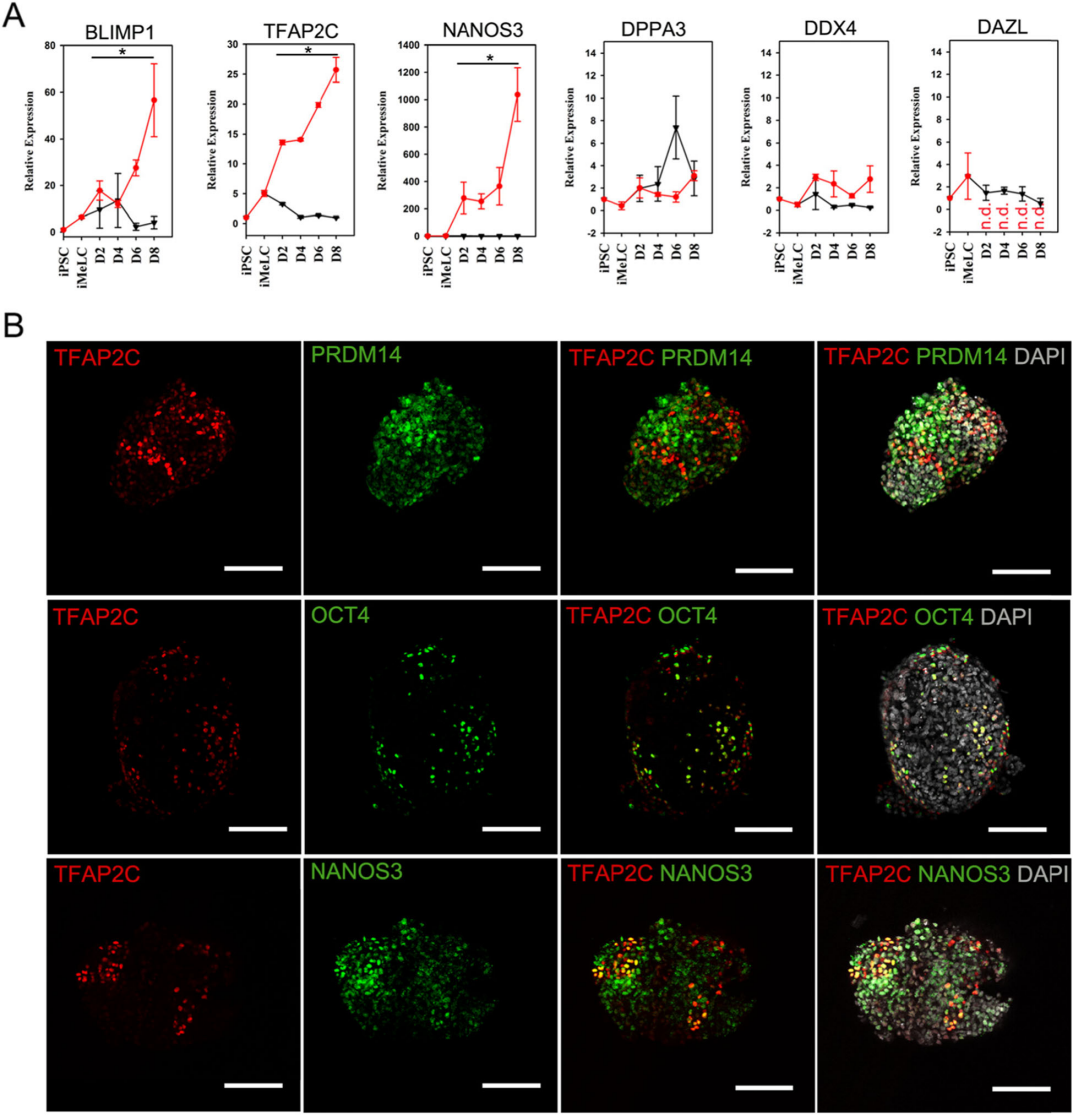
Expression analysis of germ cell specific genes during hPGCLC specification process. **a** Expression dynamics of germ cell specific genes during hPGCLC specification process, including iPSCs, iMeLCs, and the whole floating embryoids at day 2, 4, 6, and 8 of induction, as measured by qRT-PCR. Relative expression levels are shown with normalization to housekeeping gene GAPDH. Error bars indicate mean ± SD of three independent experiments. Red squares indicate values for embryoids exposed to cytokine stimulation; black triangles indicate values for embryoids formed spontaneously without cytokine stimulation. *p < 0.05 vs. the iPSC groups. n.d., not detected. **b** Immunofluorescence analysis for the expression of TFAP2C, PRDM14, OCT4, and NANOS3 in day 4 embryoids derived from hiPSCs. Scale bars, 100 μm

We established hiPSC lines from dermal fibroblasts of three male volunteers using a method published before [13]. The hiPSCs displayed typical hESC morphology and were positive for pluripotency markers, including OCT4, SOX2, and SSEA (Fig. 1a, b). After pre-induction, the hiPSCs were differentiated into flat iMeLCs with distinct cell borders, which were also positive for pluripotency markers (Fig. 1a, c). For PGCLC induction, the differentiating cells were maintained under a floating culture condition and aggregated to form embryoids (Fig. 1a). We analyzed the expression of PGC genes, including BLIMP1, TFAP2C, NANOS3, DPPA3, DDX4, and DAZL, during the differentiation process. The hiPSCs and hESCs showed no or low expression of the PGC genes, which remained at low levels after pre-induction, except for BLIMP1and TFAP2C, exhibiting slightly increased levels at iMeLC stage (Fig. 2a and Supplemental fig. 1 A). Under the stimulation for PGCLC induction, the embryoids differentiated from hiPSCs initiated significant upregulation of the early PGC genes (BLIMP1, TFAP2C, and NANOS3, *p* < 0.05), but maintained low or no levels of DPPA3 and late PGC genes (DAZL and DDX4) (Fig. 2a). However, we observed that the embryoids from hESCs increased the expression of NANOS3 and DPPA3 from day 4 of PGC induction (Supplemental fig. 1 A). Immunofluorescence analysis demonstrated that the expression of TFAP2C coincided with PRDM14, OCT4, and NANOS3 in day 4 embryoids (Fig. 2b). These findings indicate that the hiPSCs are capable of early PGC fate following PGCLC induction in vitro.

### Efficiency of PGCLC induction in vitro

**Fig. 3:**
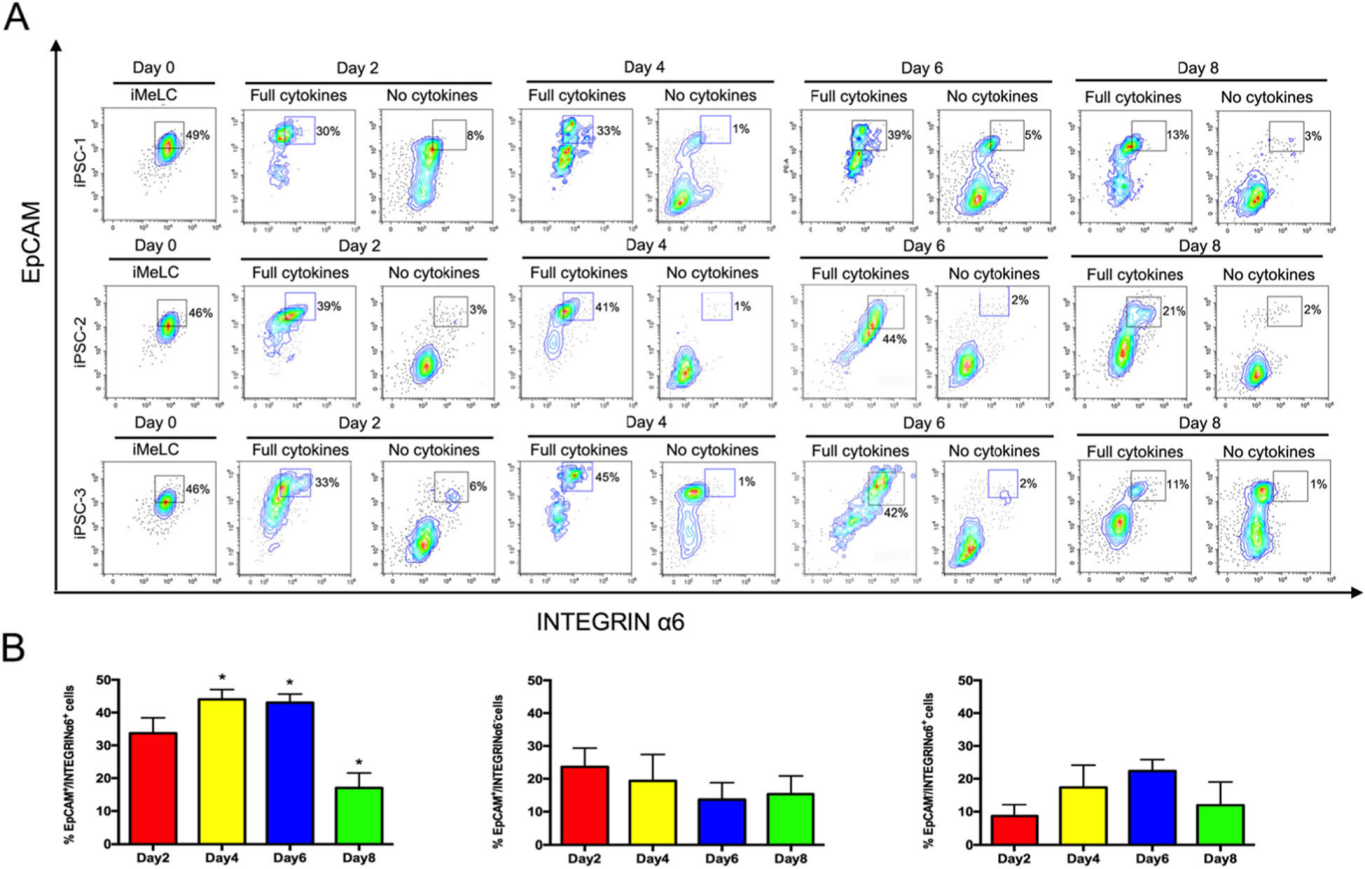
Efficiency of PGCLC induction from iPSCs in vitro. **a** FACS analysis by EpCAM and INTEGRINα6 expression of cells during hPGCLC induction (until day 8) from hiPSCs by BMP4, LIF, SCF, and EGF (left) or by no cytokines (right). Boxed areas indicate EpCAM/INTEGRINα6 double-positive cells with their percentages. **b** Percentage of cells double positive for EpCAM/INTEGRINα6, only positive for EpCAM and only positive for INTEGRINα6 in day 2, 4, 6 and 8 floating embryoids determined by FACS, respectively. Error bars indicate mean ± SD of three independent experiments. *p < 0.001 vs. the day 2 groups

Recently, EpCAM and INTEGRINα6 were identified as surface markers of hPGCLCs in embryoids [11]. We performed fluorescent-activated cell sorting (FACS) analysis for the differentiated cells by these two markers. The results showed that the percentage of EpCAM/INTEGRINα6 double-positive cells in iMeLCs (day 0) was up to 46~49% without obvious segregation, but the germ cell fate was not activated at this point and these cells were not PGCLCs. As early as day 2 of PGCLC induction, the differentiating cells in embryoids began to segregate into two different populations. The EpCAM/INTEGRINα6 double-positive proportion was increased progressively until day 6, resulting in around 39~44% of double-positive putative hPGCLCs, and the two subpopulations became more distinct and persisted until day 8 of induction, albeit with reduced ratios at day 8 (Fig. 3 and Supplemental fig. 1 B).

### Multi-lineage gene expression dynamics during hPGCLC induction process

**Fig. 4:**
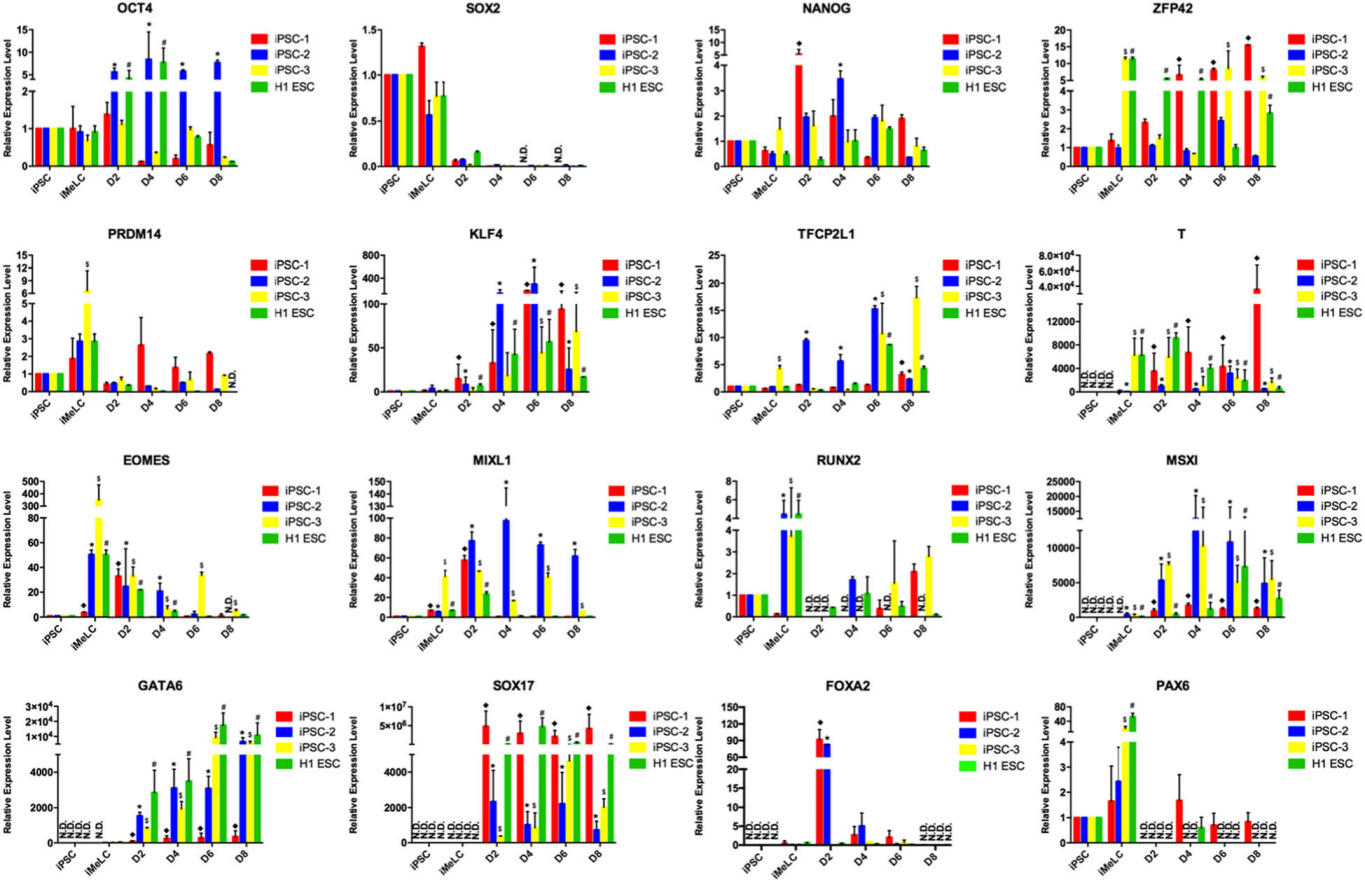
Multi-lineage gene expression analysis during hPGCLC specification process. Relative expression levels of genes in hiPSCs, iMeLCs, and the whole floating embryoids were measured by qRT-PCR and shown with normalization to housekeeping gene GAPDH. Error bars indicate mean ± SD of three independent experiments. ^◆^*p* < 0.05 vs. the iPSC-1 group, *p < 0.05 vs. the iPSC-2 group, ^$^*p* < 0.05 vs. the iPSC-3 group, ^#^*p* < 0.05 vs. the H1 ESC group. n.d., not detected

**Fig. 5:**
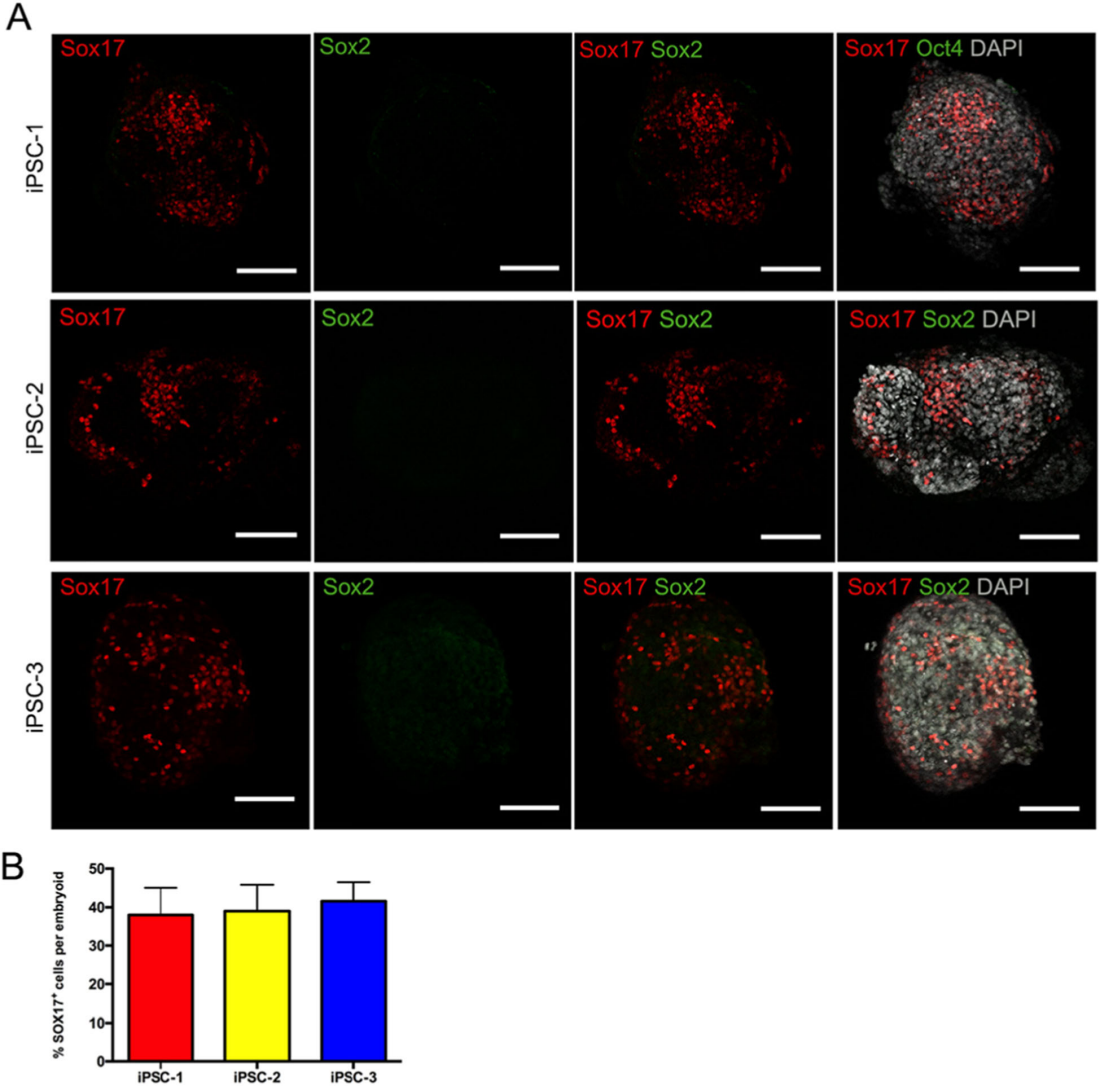
Expression analysis of SOX17 and SOX2 in PGC specification. **a** Immunofluorescence for the co-expression of SOX17 with SOX2 in day 4 embryoids derived from hiPSCs. Scale bars, 100 μm. **b** Quantification of the SOX17 positive cells in day 4 embryoids. Error bars indicate mean ± SD of five independent experiments

We also detected the expression dynamics of pluripotency and embryonic lineage genes during the PGCLC induction process by qPCR (Fig. 4). Overall, the embryoids derived from all lines upregulated or remained similar levels of key pluripotency genes, OCT4 and NANOG, but became negative for SOX2. Compared to undifferentiated cells, the differentiating cells exhibited high levels of genes associated with naive pluripotency such as ZFP42, KLF4, and TFCP2L1, whereas the expression of PRDM14, which plays an important role in the mouse PGC specification [3], did not change evidently after induction. For the embryonic lineage genes, we observed that hiPSCs showed low or no expression of genes involved in endoderm (GATA6, SOX17, and FOXA2), mesoderm (EOMES, T, MSXI, RUNX2, and MIXL1), and ectoderm (PAX6) development. After pre-induction, the differentiating cells upregulated most of the mesoderm genes (EOMES, T, MSXI, RUNX2, and MIXL1) and one ectoderm gene (PAX6) to some extent, but still showed a lack of endoderm gene expression. These results were consistent with the designated identity of iMeLCs by Sasaki et al. [11]. In contrast to iMeLCs, the differentiating embryoids downregulated mesoderm genes RUNX2 and EOMES and ectoderm gene PAX6. In mice, WNT/BMP signaling stimulates the upregulation of T expression, a key mesodermal factor for the onset of mPGC specification [14]. Here, the expression of T exhibited modest upregulation in PGCLCs, although it may not play a major role in hPGCLC specification [12]. Recently, it has been reported that WNT signaling activates EOMES at iMeLC stage to induce the expression of SOX17, a critical driver of hPGC fate, at PGCLC stage [7, 12]. Particularly, we noted that the hPGCLCs decreased the expression of EOMES and increased the expression of SOX17, compared with iMeLCs. Additionally, immunofluorescence analyses of the day 4 embryoids validated the expression of SOX17 and the repression of SOX2 in PGCLCs (Fig. 5 and Supplemental fig. 1 C), which were identical to the observation in human embryonic PGCs [15, 16].

## Discussion

Here, we differentiated hiPSCs into hPGCLCs in vitro and analyzed the expression dynamics of multi-lineage genes during the differentiation process. While the PGC fate regulator genes were activated, the embryonic lineage genes in the embryoids present complex expression patterns after induction, providing insights for future studies on human early embryo development.

From fertilization to blastocyst formation, mouse and human pre-implantation embryo development are morphologically similar; however, timing and molecular details of important developmental events occurring at this stage may differ, like zygotic genome activation (ZGA), X-inactivation, signaling requirement for pluripotency, and blastocyst lineage specification [17–20]. From implantation to gastrulation, human and mouse embryos become structurally distinct, and the germ layer formation takes place at this stage [18]. In mice, PGC specification occurs at the posterior epiblast before gastrulation [2], which is supported by the complicated signaling interactions between embryonic and extra-embryonic tissues in the egg cylinder [21]. However, less is known about the origin and specification mechanism of hPGCs in the early post-implantation human embryos. It is demonstrated that BMP signaling is very likely to be conserved for PGC specification in mammals, including human [7, 11, 22].

Until recently, several studies reconstituted hPGC specification from hESCs in vitro and defined the signaling and transcriptional programs for human germ cell specification in vitro. In brief, WNT signaling (ACTIVIN A) activates EOMES to induce the expression of SOX17, which is a critical regulator of hPGC fate and is upstream of BLIMP1 [7, 11, 12]. SOX17 upregulates BLIMP1 and, potentially, endoderm genes. TFAP2C is initially activated independently from SOX17 through the BMP signaling. SOX17 and TFAP2C work together to establish the hPGCLC transcriptional program in competent cells upon induction by BMP signaling. The expression of downstream BLIMP1 represses SOX17-induced endoderm genes, BMP- and WNT-induced mesoderm genes, as well as other somatic genes [7]. Hypothetically, this signaling and transcription model for hPGC specification would occur in the nascent amnion in early post-implantation embryos.

Additionally, PRDM14 is expressed in the differentiating cells at a relatively low level and the expression of T is increased after BMP induction, whereas the role of PRDM14 and T in hPGC specification remains to be clarified. Notably, SOX2 is suppressed as soon as the induction starts. BMP signaling and BLIMP1 expression may contribute to the rapid downregulation of SOX2 during hPGC specification [23, 24]. Moreover, the embryoids derived from hiPSCs show different regulation for the three germ layer genes, suggesting that only parts of the hiPSCs respond to the BMP signaling to initiate the germ line specification; the other cells may still get into somatic cell fate.

## Conclusions

In conclusion, we demonstrated that during PGCLC induction process in vitro, the differentiating embryoids not only activated the PGC-related genes, but also displayed complex regulation of pluripotency genes and multi-lineage genes. Nevertheless, further studies on human and non-human primate embryo development, especially for high-throughput studies, are needed to explore the complicated regulation networks of hPGC specification.

## Supplementary information

**Additional file 1: Figure S1.** Differentiation of hESCs into PGCLCs in vitro. (A) Expression dynamics of germ cell specific genes during hPGCLC specification process, including hESCs, iMeLCs, and the whole floating embryoids at day 2, 4, 6 and 8 of induction, as measured by qRT-PCR. Relative expression levels are shown with normalization to housekeeping gene GAPDH. Error bars indicate mean ± SD of three independent experiments. Red squares indicate values for embryoids exposed to cytokine stimulation; black triangles indicate values for embryoids formed spontaneously without cytokine stimulation. *p < 0.05 vs. the hESC groups. n.d., not detected. (B) FACS analysis by EpCAM and INTEGRINα6 expression of cells during hPGCLC induction (until day 8) from hESCs by BMP4, LIF, SCF, and EGF (left) or by no cytokines (right). Boxed areas indicate EpCAM /INTEGRINα6 double positive cells with their percentages. (C) Immunofluorescence for the co-expression of SOX17 with SOX2 in day 4 embryoids derived from hiPSCs. Scale bars, 100 μm.

## Data Availability

All relevant data are available from the authors upon reasonable request.

## Abbreviations

PGCs: Primordial germ cells
BMP: Bone morphogenetic protein
iPSCs: Induced pluripotent stem cells
PGCLCs: PGC-like cells
PRDM1: PR domain zinc finger protein 1
TFAP2C: Transcription factor AP-2 gamma
ESCs: Embryonic stem cells
EpiLCs: Epiblast-like cells
iMeLCs: Incipient mesoderm-like cells
ZGA: Zygotic genome activation
FACS: Fluorescent-activated cell sorting
