# Supplementary material for: Analysis of multi-lineage gene expression dynamics during primordial germ cell induction from human induced pluripotent stem cells
Source: Stem Cell Res Ther. 2020 Mar 4;11:100. doi: 10.1186/s13287-020-01620-y (PMC7055065; doi:10.1186/s13287-020-01620-y)
Supplement: Supplementary file 1 — Additional file 1: Figure S1. Differentiation of hESCs into PGCLCs in vitro. (A) Expression dynamics of germ cell specific genes during hPGCLC specification process, including hESCs, iMeLCs, and the whole floating embryoids at day 2, 4, 6 and 8 of induction, as measured by qRT-PCR. Relative expression levels are shown with normalization to housekeeping gene GAPDH. Error bars indicate mean ± SD of three independent experiments. Red squares indicate values for embryoids exposed to cytokine stimulation; black triangles indicate values for embryoids formed spontaneously without cytokine stimulation. *p < 0.05 vs. the hESC groups. n.d., not detected. (B) FACS analysis by EpCAM and INTEGRINα6 expression of cells during hPGCLC induction (until day 8) from hESCs by BMP4, LIF, SCF, and EGF (left) or by no cytokines (right). Boxed areas indicate EpCAM /INTEGRINα6 double positive cells with their percentages. (C) Immunofluorescence for the co-expression of SOX17 with SOX2 in day 4 embryoids derived from hiPSCs. Scale bars, 100 μm. [file 13287_2020_1620_MOESM1_ESM.docx]

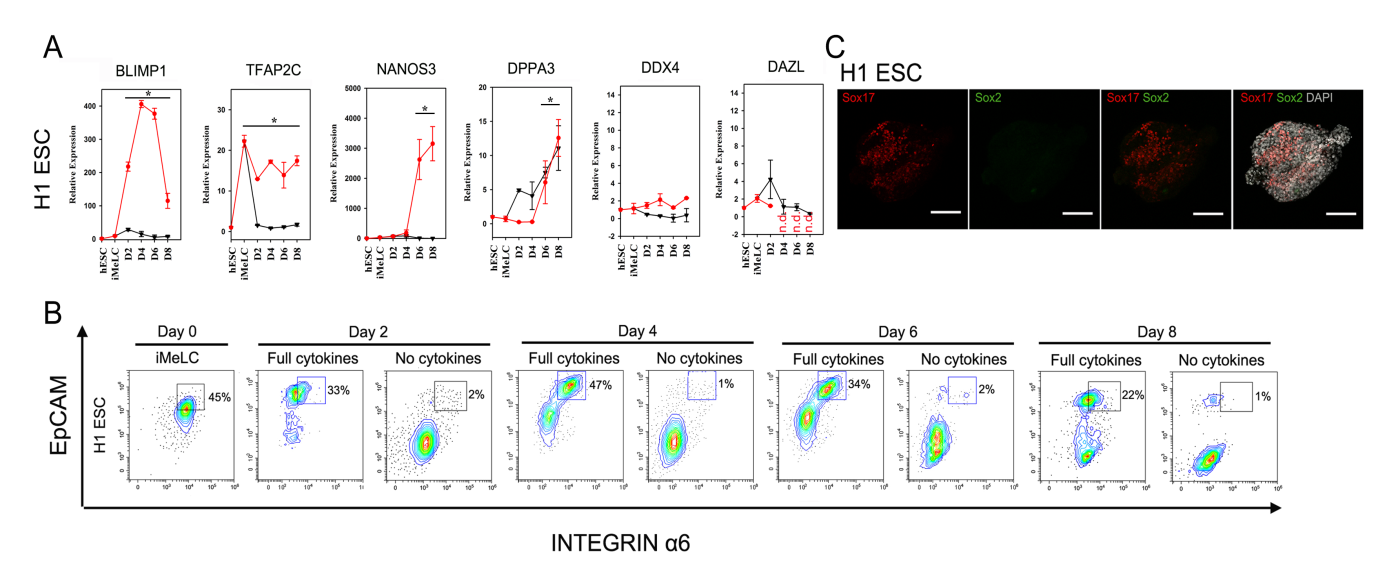


**Supplemental figure 1** Differentiation of hESCs into PGCLCs *in vitro*. (A) Expression dynamics of germ cell specific genes during hPGCLC specification process, including hESCs, iMeLCs, and the whole floating embryoids at day 2, 4, 6 and 8 of induction, as measured by qRT-PCR. Relative expression levels are shown with normalization to housekeeping gene GAPDH. Error bars indicate mean ± SD of three independent experiments. Red squares indicate values for embryoids exposed to cytokine stimulation; black triangles indicate values for embryoids formed spontaneously without cytokine stimulation. **p*<0.05 vs. the hESC groups. n.d., not detected. (B) FACS analysis by EpCAM and INTEGRINα6 expression of cells during hPGCLC induction (until day 8) from hESCs by BMP4, LIF, SCF, and EGF (left) or by no cytokines (right). Boxed areas indicate EpCAM /INTEGRINα6 double positive cells with their percentages. (C) Immunofluorescence for the co-expression of SOX17 with SOX2 in day 4 embryoids derived from hiPSCs. Scale bars, 100 μm.
